# Comparison and optimization of ten phage encoded serine integrases for genome engineering in *Saccharomyces cerevisiae*

**DOI:** 10.1186/s12896-016-0241-5

**Published:** 2016-02-09

**Authors:** Zhengyao Xu, William R. A. Brown

**Affiliations:** School of Life Sciences, Queens Medical Centre, Nottingham University, Nottingham, NG7 2UH UK

**Keywords:** Phage encoded serine integrases, Cassette exchange, *Saccharomyces cerevisiae*, Genome modification

## Abstract

**Background:**

Phage-encoded serine integrases, such as ϕC31 integrase, are widely used for genome engineering but have not been optimized for use in *Saccharomyces cerevisiae* although this organism is a widely used organism in biotechnology.

**Results:**

The activities of derivatives of fourteen serine integrases that either possess or lack a nuclear localization signal were compared using a standardized recombinase mediated cassette exchange reaction. The relative activities of these integrases in *S. cerevisiae* and in mammalian cells suggested that the major determinant of the activity of an integrase is the enzyme itself and not the cell in which it is working. We used an inducible promoter to show that six integrases were toxic as judged by their effects upon the proliferative ability of transformed yeast. We show that in general the active phage-encoded serine integrases were an order of magnitude more efficient in promoting genome integration reactions than a simple homologous recombination.

**Conclusions:**

The results of our study allow us to identify the integrases of the phage ϕBT1, TP901 ~ nls, R4, Bxb1, MR11, A118, ϕK38, ϕC31 ~ nls, Wβ and SPBC ~ nls as active in *S. cerevisiae* and indicate that vertebrate cells are more restricted than yeast in terms of which integrases are active.

**Electronic supplementary material:**

The online version of this article (doi:10.1186/s12896-016-0241-5) contains supplementary material, which is available to authorized users.

## Background

Site-specific recombinases are a class of protein that promote reciprocal and conservative recombination between two specific sequences and are widely used for genome modification. The most commonly used site-specific recombinases, *Cre* [1] and *Flp* [2], belong to the tyrosine recombinase class of site-specific recombinases and promote reversible recombination between two identical 34 bp sequences in the absence of accessory proteins. The reversible nature of these reactions has meant that although they are ideal for promoting deletions they are less suited for the insertion of DNA into the genome. The second class of site-specific recombinase are the serine recombinases. This class of protein includes recombinases with a wide variety of different properties [3]. Some such as the γδ, Hin and Gin recombinases promote reactions between identical sites but do so with topological specificity as a result of the participation of accessory proteins. Others, that are members of the so called “large serine recombinase family” promote unidirectional reactions between two different sequences, each about 50 bp in length, in the absence of accessory proteins [4]. Integrases of many bacteriophages of the actinomycetes, including that of the *Streptomyces* phage ϕC31 belong to the “large serine recombinase family” [5]. The functional autonomy of the serine integrases together with their unidirectional nature has meant that they are an ideal complement to *Cre* and *Flp,* and several such integrases including those of the BxB1, ϕC31 and R4 phages are widely used for genome integration in metazoan systems such as humans, mice and *Drosophila melanogaster* [6].

*Saccharomyces cerevisiae* is a key organism in biotechnology whose utility rests both on features of its biology such as the ability to ferment sugar but also on a wide range of tools that allow its genetic manipulation. Although the *Cre* recombinase [1] has been used in *S. cerevisiae* we are unaware of any systematic attempt to establish the utility of the serine integrases in *S. cerevisiae*. Previous work in mammalian cells [7] however has shown that the serine integrases vary widely in their activities and that BxB1 and ϕC31 integrases are particularly active. In light of the central importance of *S. cerevisiae* in bio-technology we wanted first of all to determine the rank order of activities of different integrases in promoting integration into the genome of *S. cerevisiae*. Previous experiments using ϕC31 integrase in eukaryotic cells have also shown a variable requirement for a nuclear localization signal (NLS). In chicken DT40 cells the requirement is absolute [8], in mouse 3 T3 cells the activity of the enzyme was increased by 60 % as a result of addition of a C terminal NLS [9] while in *Drosophila melanogaster* there appears to be no requirement at all [10]. In light of these observations our second question was therefore to assess the requirement of the different integrases for an NLS in *S. cerevisiae*. Finally previous work in mammalian cells [7] has shown that the serine integrases were toxic to various degrees. Our third objective was therefore to establish the toxicities of the different integrases in both NLS modified and unmodified form in *S. cerevisiae*. In summary we set out to establish a set of integrases for use in *S. cerevisiae* and to define the optimal conditions for their use. The enzymes that we have chosen for these investigations (ϕC31 [4], Bxb1 [11], ϕBT1 [12], ϕC1 [13, 14], MR11 [15], TP901-1 [16], R4 [17], A118 [14], and ϕRV [14]. Bibb et al. [18], TG1, ϕ370.1 [19], Wβ [20], SPBC and ϕK38 ) are those that we previously studied in human fibroblasts and mouse ES cells [7] and thus our results allow us to address the question as to the degree to which enzymes established as optimal in one type of eukaryotic cell are likely to work well in another and the extent to which the activities of these integrases show species specific effects.

## Results and discussion

### Assaying unidirectional phage integrases in *S. cerevisiae*

**Fig. 1:**
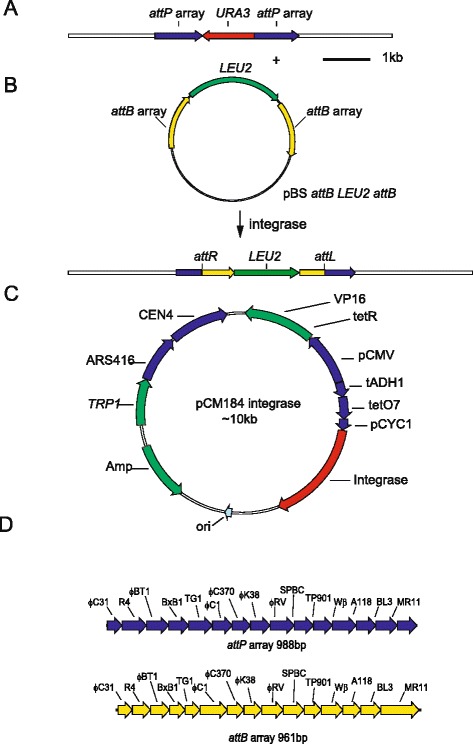
Sequence organization of the DNA used to assay integrase activity in *Saccharomyces cerevisiae.* **a** The structure of the *URA3* containing integrase assay construct integrated at the *LEU2* locus. **b** The structure of the donor plasmid used to assay integrase mediated cassette exchange and the product of site-specific recombination between this plasmid and the *URA3* containing integrase assay construct. **c** The generic structure of the plasmid used to inducibly express the integrases. The plasmid is based upon the “Tet-off” vector pCM184. **d** The sequence organization of the *attP* and *attB* arrays used in the target and donor plasmids used to assay integrase mediated cassette exchange

The approach that we took to determining the relative activities of the different integrases in *S. cerevisiae* is analogous to the recombinase mediated cassette exchange approach that we used to assay the activities of these same integrases in vertebrate cells [7]. In this approach we use a single construct to assay all of the integrases; this assay construct contained a counter selectable marker gene, *URA3,* flanked on either side by identical arrays of *attP* sites (Fig. 1a, d) for each of the integrases that we wanted to assay. The sequences of the attachment sites are given in Additional file 1: Table S1. This construct was integrated at the *LEU2* locus by homologous recombination. We wanted to use a regulated promoter to drive expression of the different integrases in *S. cerevisiae* because such a promoter would enable us to balance the level of expression and any potential toxicity. Several inducible promoters are available for use in *S. cerevisiae*. These potentially would enable one to determine the relationship between the activity, the toxicity and the amount of the different integrases. The most commonly used regulated expression vector is based upon the GAL1-10 system [21]. This promoter allows a 1000 fold induction but is glucose repressible and thus requires a change of carbon source prior to induction with galactose with the concomitant risk of pleiotropic effects. Other systems such as the MET3 [22] and PHO5 promoters [23] are also unsuitable because they either have a low range of induction ratios or also require nutritional changes prior to induction. We have therefore used the tTA-tetO system developed by Herrero and colleagues [24]. This system uses the standard tTA repressor in a fusion with a viral VP16 transcriptional activator [25]. This fusion is a transcriptional activator that fails to bind the tetracycline response element in the presence of tetracycline and as such is a so-called “Tet-off” system [25, 26]. The plasmid allows for 1,000 fold induction of expression upon removal of tetracycline analogue doxycycline (DOX) [24]. Thus induction can be achieved simply by washing the DOX out of the medium without any other change in its composition and as such is ideal for our purposes. The fact that expression is induced by removal of the DOX is sometimes confusing and so we have simplified the presentation of the data by colour coding the induced and un-induced states by green and red respectively.

We expressed a particular integrase either with or without a nuclear localization signal from a codon optimized integrase gene as in the cells using the “Tet-off” inducible expression vector pCM184 (Fig. 1c). We measured integrase activity by the replacement of *URA3* by a second selectable marker gene; *LEU2* that had been flanked by two arrays of *attB* recombination sites (Fig. 1b, d and Additional file 1: Table S1 for the sequences of the attachment sites). We used this system to assay the activities of the different integrases by comparing the efficiencies with which the different integrases promoted the cassette exchange reaction as determined by the number of colonies recovered after transformation and plating onto medium lacking leucine.

The use of a single pair of assay plasmids in this way allowed us to assay all the integrases that had sites in both of the arrays using just a single clone of cells containing the genomic assay construct. Thus although the design of the system that we are using in *S. cerevisiae* is similar to the one that we used in mammalian cells*,* the ease of gene targeting and the availability of simple inducible expression system in *S. cerevisiae* allowed for greater control of the structure of the target locus and of the level of expression of the integrases than was possible in mammalian cells.

### Toxicity of unidirectional phage integrases in *S. cerevisiae*

**Fig. 2:**
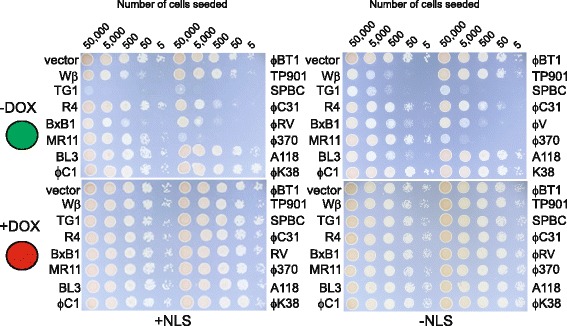
Assaying toxicity of fifteen phage integrases in *S. cerevisiae.* Serial ten fold dilutions of yeast expressing a native integrase or an integrase tagged at the C terminus with a nuclear localization signal were plated on SD-Trp agar starting at an initial inoculum of 5 x 10^4^ in the presence or absence of doxycycline and cultured for three days before recording growth

Previous work in mammalian cells has shown that phage integrases are often toxic and that different integrases are toxic to varying degrees [7]. The short doubling time of *S. cerevisiae*, ease of manipulation and the availability of a tightly regulated expression vector allowed us to investigate this question of integrase toxicity with greater sophistication than was possible in mammalian cells where toxicity was noted simply by a failure to recover stably transfected clones that expressed the integrase. In order to do this we introduced the expression construct for each of the different integrases as both native and NLS modified forms into the yeast cells in the presence of DOX (integrase expression: “off”) . We isolated transfected clones and then placed different numbers of cells expressing either the native integrase or the integrase tagged with a NLS under conditions in which expression of the integrase was either on (-doxycycline (-DOX); green circle) or off (+doxycycline (+DOX); red circle) and compared the seeding efficiency of the cells containing the integrase expression vector with a clone that contained an empty expression vector (Fig. 2). The results demonstrated that cells expressing the integrases Wβ, TG1, SPBC, BxB1, ϕC31, ϕRV and ϕ370 showed reduced seeding efficiencies regardless of the presence of a NLS in the absence of DOX (integrase expression: “on”) and so we concluded that they were toxic when expressed at high levels. We also noted that possession of a NLS promoted toxicity in the case of the TG1 and BxB1 recombinases. The simplest explanation for these results was that the integrase in question was toxic because it was interacting in some way with the nuclear genome. Finally in the case of the Wβ and BL3 recombinases the effect of the NLS was to reduce the toxicity of the integrase. This maybe because these particular integrases do not depend upon the NLS to enter the nucleus and that the NLS compromises the ability of these integrases to interact with the genome; perhaps by reducing binding of the integrase to ectopic recombination sites. In summary these results emphasized that the integrases needed to be regarded individually with respect to their toxicities and suggests that the optimal conditions for using the different integrases may require that the toxicity and the level of expression required for optimum activity be balanced.

### Comparing the activities of unidirectional phage integrases in *S. cerevisiae* using arrays of recombination sites

**Fig. 3:**
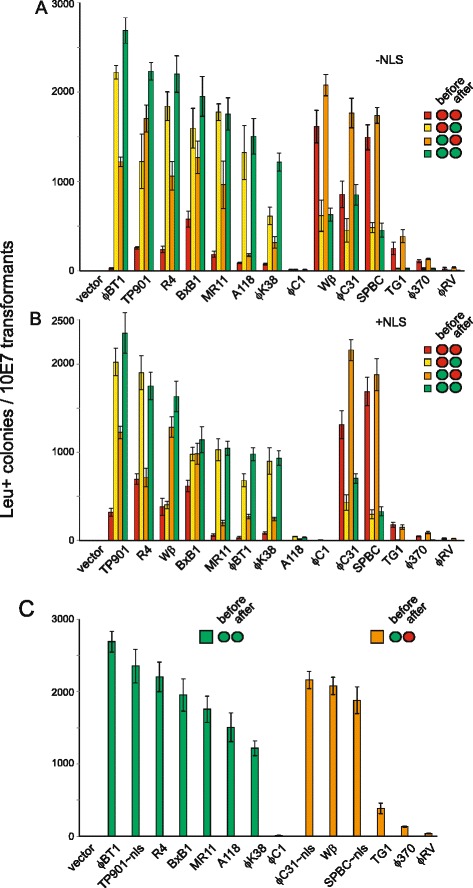
Assaying activity of fourteen phage integrases in *S. cerevisiae.* Strains expressing containing the integrase assay plasmid (Fig. 1c) and expressing the indicated integrase were transformed with the *LEU2* donor plasmid (Fig. 1b) and selected for leucine prototrophy. The number of leucine prototrophs recovered following transformation of 10^7^ cells is indicated and as the negative control shows reflects the activities of the integrases. Each integrase was assayed under four different patterns of expression with respect to the transformation. Each transformation was repeated four times with the mean and sample standard deviation indicated. The four conditions correspond to either induction or non-induction of integrase expression either before or after transformation and this is indicated by the traffic light cartoon in the top right hand corner of each part of the figure. (Non-induction (red) is when the cells are grown in the presence of Doxycycline which represses the Tet promoter and induction (green) corresponds to growth in the absence of Doxycycline). **a** The activity of each integrase lacking a nuclear localization signal. Integrases judged to be toxic on the basis of the analyses shown in Fig. 2 are grouped on the right hand side of the panel. **b** The activity of the integrases with a C-terminal nuclear localization signal and, as in A, integrases judged to be toxic on the basis of the analyses shown in Fig. 2 are grouped on the right hand side of the panel. **c** A summarizes the different integrases according to the rank order of the activities of their optimally active forms and whether they were toxic or not. As in A and B, integrases judged to be toxic are grouped on the right hand side of the panel

We used the marker swap strategy described above to assay the activities of the integrases as a function of the presence of a nuclear localization signal and to determine whether activity was affected by inducing expression of the integrase before or after the transformation event (Fig. 3). In preliminary experiments we also assayed the activity of the BL3 integrase but were unable to produce results that were consistent between experiments and therefore we did not continue with the analysis. We confirmed that the numbers of leucine prototrophic colonies recovered in these experiments accurately reflected cassette exchange activity by PCR and sequencing across the recombination breakpoints within the flanking cassette DNA. For each recombinase we analysed a total of 40 events by PCR (Additional file 1; Figure 1 shows the data obtained for the BxB and ϕC31 integrase recombinants as an example of our results) and checked 15 *attL* and *attR* sites of each integrase by sequencing (for examples see Additional file 1; Figure 2) (Table 1). This sequencing confirmed that the reaction had occurred in a reciprocal and conservative fashion and without site damage. We also checked for loss of the *URA3* marker for ϕC31, BxB1, TP901, Wβ, RV and ϕC1 integrases by testing for growth on uracil deficient medium. In all cases the results of the PCR and growth tests were consistent. Finally we confirmed that these analyses were reliable by checking four recombination events generated by BxB1 integrase by gel electrophoresis and filer hybridization. This confirmed that the sequence organization of the locus before and after cassette exchange was as predicted (Additional file 1; Figure 3).

**Table 1:** Assaying integrase mediated site-specific recombination in yeast *S.cerevisiae*

Integrase	PCR for site specific recombination *attL* and *attR* PCR	Sequence for attL (correct sample /checking number)	Sequence for attR (correct sample/checking number)
ΦBT1	9/10^+^; 10/10^+^; 10/10^−^; 10/10^−^	15/15	15/15
Wβ	10/10^+^; 10/10^+^; 10/10^−^; 10/10^−^	15/15	15/15
TP901	10/10^+^; 10/10^+^; 10/10^−^; 10/10^−^	15/15	15/15
TG1	9/10^+^; 9/10^+^; 9/10^−^; 9/10^−^	15/15	15/15
SPBC	10/10^+^; 10/10^+^; 10/10^−^; 10/10^−^	15/15	15/15
R4	9/10^+^; 8/10^+^; 8/10^−^; 9/10^−^	15/15	15/15
ΦC31	10/10^+^; 10/10^+^; 10/10^−^; 10/10^−^	15/15	15/15
BxB1	10/10^+^; 10/10^+^; 10/10^−^; 10/10^−^	15/15	15/15
RV	2/10^+^; 2/10^+^; 22/95^−^; 2/10^−^	15/15	15/15
MR11	9/10^+^; 10/10^+^; 10/10^−^; 10/10^−^	15/15	15/15
Φ370	8/10^+^; 7/10^+^; 9/10^−^; 9/10^−^	15/15	15/15
A118	6/10^+^; 5/10^+^; 7/10^−^; 7/10^−^	15/15	15/15
ΦC1	0/50^+^; 0/50^+^; 0/50^−^; 0/50^−^	NA	NA
ΦK38	9/10^+^; 8/10^+^; 10/10^−^; 9/10^−^	15/15	15/15

Table 1 PCR was carried out following transformation to measure the proportion of Leu^+^ clones that had undergone recombinase mediated cassette exchange. At least 10 clones were analysed from the strains containing the nls + integrase or the nls- integrase. (+ or – superscript respectively). Fifteen PCR products derived from each type of strain were confirmed by DNA sequencing as corresponding to reciprocal and conservative site-specific recombinants. In the case of RV integrase this involved screening additional Leu^+^ Ura^−^colonies by PCR for those that had arisen by site-specific recombination

The results illustrated in Fig. 3 showed that the level of cassette exchange was sensitive to whether the integrase had been induced. However whether induction increased or decreased the detected recombination activity depended upon whether the integrase was toxic as determined by the experiments described above. Optimal activity of non-toxic integrases was observed when integrase expression was induced both before and after expression while for most of those integrases judged to be toxic, induction of expression after transformation reduced the number of *LEU2*^+^ transformants that were recovered. It is striking that the three of the toxic integrases (ϕC31, SPBC and Wβ) showed significant levels of activity in the absence of any induction (red bars in Fig. 3a and b) in contrast to the non-toxic integrases. It is not clear why this is so although one possibility is that these integrases are in fact very active and their detectable activities are limited by toxicity arising as a result of interaction with yeast genomic sequences that bear some similarity to their respective *attB* or *attP* sites. For most of the integrases the presence of a NLS (Fig. 3b) had little effect upon the detectable activity although in general the effect of a NLS was to reduce it. The only integrases showing exceptions to this pattern were the TP901 and the ϕC31 integrases whose detectable activities were slightly increased by the addition of a NLS. In vertebrate cells we [27] and others [28] have observed that site damage can occur during site-specific recombination. However all of the sequenced *attL* and *attR* sites indicated that the site-specific recombination was both reciprocal and conservative and correspondingly that no damage to any of the sites had occurred. Given that we analyzed a total of 300 *attL* and *attR* sites these results suggest that site damage accompanying site specific recombination mediated by serine integrases occurs rarely in *S. cerevisiae*. In conclusion the ϕBT1, TP901, R4, Bxb1, MR11, A118, ϕK38, ϕC31, Wβ and SPBC are all providing useful levels of activity as assayed using the array based marker swap approach. The activities of the optimal form of each of the 14 different integrases assayed using optimal induction conditions are ranked in Fig. 3c. We carried out unpaired two sided t- tests assuming unequal variances to assess the significance of the differences between the activities and observed that the differences between the activities of integrases of adjacent rank were not significant but that differences between integrases separated by more than two or more rank orders were significant at the *P* < 0.05 level; for example the activities of the ϕBT1 and TP901 ~ NLS integrases were not significantly different but the activities of the ϕBT1 and R4 integrases were significantly different. In order to obtain the optimal level of activity for any particular integrase however our experiments show that one needs to use the induction conditions appropriate to the toxicity of the integrase. Thus for the integrases that are listed on the left hand side of Fig. 3c they are most active if integrase expression is induced both before and after transformation with the donor plasmid while for the toxic integrases listed on right hand side of Fig. 3c integrase expression should only be induced prior to expression.

### Comparing the activities of unidirectional phage integrases in *S. cerevisiae* using single pairs of inverted recombination sites

**Fig. 4:**
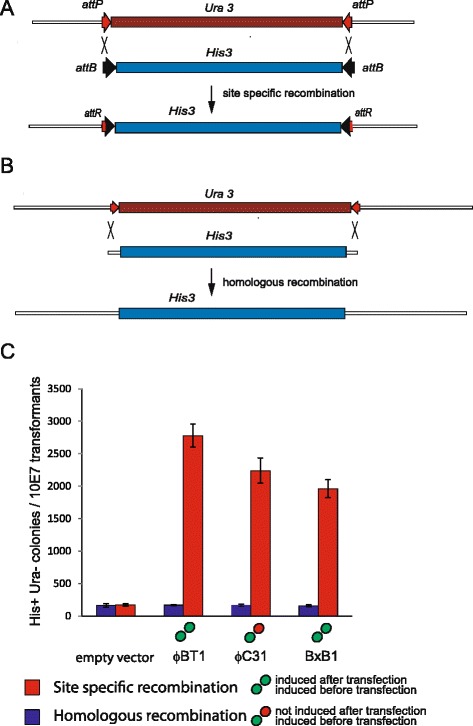
Assaying three phage integrases in *S. cerevisiae* using single target sites. **a** The assay for site specific recombination using single sites integrated into the *LEU2* locus. Recombinants were assayed by uptake of the *LEU2* gene and loss of the *URA3* gene. **b** The assay for homologous recombination at *LEU2*. There was a total of 60 bp of homology with the *LEU2* locus at either end of the *HIS3* gene. **c** A summary of the activities of the optimally active forms of the ϕBT1, ϕC31 and BxB1 integrases according to their respective rank orders compared with the results of the homologous recombination assay carried out in the same experiment. All assays were carried out four times with the mean and standard deviations indicated

In the experiments described above we used *URA3* and *LEU2* genes flanked by arrays of multiple attachment sites because this approach allowed us to use a single assay construct and a single transformed strain containing the assay construct to assay all of the integrases. This increased the through-put of the assay. However this is not the way in which the integrases are likely to be used routinely where typically one will want to build constructs containing marker genes with single sites using PCR. We therefore needed to establish whether the results obtained from the high through-put approach were informative when single sites were used. We therefore built reporter strains specific for individual integrases in which the *URA3* counter selectable marker gene was flanked by *attP* sites arranged in an inverted orientation, targeted this to the *LEU2* locus and then measured site specific recombination by the cognate integrase using displacement of the *URA3* marker by an *HIS3* gene flanked by *attB* sites arranged in an inverted orientation (Fig. 4a).

Genome modification in *S. cerevisiae* is often carried out using homologous recombination and we wanted to compare the integration efficiencies that we obtained as a result of site-specific recombination with those obtained using homologous recombination. We therefore also designed a homologous recombination substrate that we used in conjunction with the site specific recombination assay (Fig. 4b). Therefore we flanked the *HIS3* gene with 60base pairs of homology flanking the *LEU2* coding region and used this as an alternative substrate in the reconstitution assay. We used these assays to address two questions; firstly, was the activity of the integrases in a single site assay comparable to what had been observed in the assay in which the substrates had been flanked by arrays of sites and secondly how did the activities compare with those observed using homologous recombination? In making this comparison we used three integrases whose activities we had optimized using the array based assay. The results of the experiments are summarized in Fig. 4c. We used long range PCR to confirm that the structures of the target locus before and after site-specific recombination with BxB1 and ϕC31 integrase was as predicted indicating that the assay accurately reflected site-specific recombination each (Additional file 1: Figure S4). We carried out the site-specific recombination experiments four times and on each also confirmed by PCR that all of the Leu^+^ Ura^−^ transformants had arisen by site-specific recombination (Additional file 1: Figure S5 shows the results of the analysis of the BxB1 and ϕC31 clones while all of the data are summarized in Additional file 1: Table S4). We analysed the results of the site-specific recombination experiments (Fig. 4c) for significance using two sided t tests and observed that the differences between the activities of the ϕBT1 and ϕC31 integrases were significant but that the differences between the activities of ϕC31 and BxB1 integrases were not at the level of P < 0.05. Although the rank order of integrase activities obtained using the two approaches was not identical it was observed firstly (Fig. 4c) that those integrases that were active in the array based assay were also active using single site assays and that the integration activity of the integrases was approximately an order of magnitude higher than that of homologous recombination. We therefore conclude that the array based approach was reliable in identifying which were the active integrases and thus that we have established a portfolio of integrases for use in genome modification in *S. cerevisiae* and have defined conditions that allow them to be used most efficiently.

## Conclusions

Our results firstly allow us to identify a portfolio of the following 10 integrases: ϕBT1, TP901, R4, Bxb1, MR11, A118, ϕK38, ϕC31, Wβ and SPBC as active in mediating recombinase mediated cassette exchange in *S. cerevisiae* at similar although not identical levels of activity. The rank order of their activities is as follows: ϕBT1 > TP901 ~ R4 ~ Bxb1 ~ Wβ nls ~ ϕC31nls ~ MR11 SPBCnls, A118 > ϕK38 > > TG1 > > ϕ370 > ϕRV. In contrast to the situation in vertebrate cells [7, 27, 29], we detected no site damage associated with integrase mediated site-specific recombination despite screening a total of 300 different *attL* and *attR* sites. Of the 10 usefully active integrases, four; Wβ, SPBC, BxB1 and ϕC31 were shown to be toxic and, for their most efficient use, expression should only be induced prior to transformation of the recombining substrates. Despite the toxicity observed with four of these integrases these observations can be compared with those that we obtained when studying the same set of integrases in mammalian cells. As a result of those experiments we concluded that only the ϕBT1, R4, Bxb1, ϕC31, Wβ and SPBC were active in either mouse or human cells as judged by site-specific recombinase mediated deletion of a counter selectable marker and of these only ϕBT1, R4, Bxb1 and ϕC31could be used for recombinase mediated cassette exchange [7]. Thus although there is overlap between the recombinases that can be used in yeast and the ones that can be used in mammalian cells it is also clear that yeast are less restrictive in terms of which recombinases are active in mediating both deletion and cassette exchange. The fact that toxic integrases can be used efficiently in yeast but require specific conditions however means that new integrases will need to be tested for toxicity as well as activity prior to their most efficient exploitation in yeast. Thirty four new phage integrases [30] have recently been described and shown to be active in bacteria. It will be of interest to determine what proportion of these can be used in *S. cerevisiae* and of these how many are toxic.

One observation that we have made is that these phage integrases are on average ten- fold more active than homologous recombination in their integration activity. However it is important to note that the efficiency of homologous recombination can be increased by between 100 and 1000 fold by introducing a double strand break into the target site [31] which we did not do. Thus decisions about whether to use homologous or site specific recombination as an approach for integration reactions will depend upon the experimental circumstances.

## Methods

### Plasmids and integrases

Plasmids were constructed either using T4 DNA ligase or using SLiCE [32] as described in detail in the Additional file 1. The integrase genes [7] were codon optimized for expression in mouse cells and were initially tagged at the carboxy termini with the SV40 large T antigen nuclear localization signal. Derivatives lacking this NLS were constructed by PCR. The expression plasmid pCM184 [24] was obtained from Addgene. The arrays of *attB* and *attP* sites used in the recombination assays were described in [7] and the sequences are given in Additional file 1: Table S1. All plasmids were checked by sequencing prior to use. A complete list of the plasmids (Additional file 1: Table S2) used in this work may be found in the Additional file 1 together with details of the primers used in their construction (Additional file 1: Table S3). The PCR products used in the experiment using single pairs of inverted sites were prepared as follows; the *attP* URA sequences were amplified using the primers 622 and 623, 630 and 631 and 592 and 593 for ϕBT1, BxB1 and ϕC31 respectively, the *attB* His sequences were amplified using primers 644 and 645, 642 and 643 and 646 and 647 for ϕBT1, BxB1 and ϕC31 respectively and the *HIS3* gene tagged with sequences for homologous recombination at the *LEU2* locus was amplified with primers 648 and 649. The sequences of the respective primers are given in Additional file 1: Table S3.

### Yeast methods

The experiments described in this paper used the strain T7107: *MATa leu2 ura3 trp1 ade2 his3* (a gift of Conrad Nieduszynski). Strains were either grown in YPD medium or for selections SD medium supplemented with the required auxotrophic supplements obtained from Formedium. pCM184 [24] was used as the vector for expressing the integrases . The pCM184 is a Tet-off vector, so gene expression is induced when tetracycline (Tc) or doxycycline (Dox) is removed from the culture medium. For the recombination assay, the reporter strain (ZXY1) was grown in SD-Ura-Trp medium with or without doxycycline (1 μg/ml) to select for the pCM184 plasmid until transformation. After the *attB LEU2 attB* donor plasmid was introduced into the reporter cell by transformation, recovered for the cells were grown on SD-Leu-Trp agar plates with or without doxycycline (1 μg/ml). Under these conditions only cells that have taken up the *LEU2* gene present in the donor cassette and also retain the integrase expression plasmid pCM184 (Fig. 1c) containing the *TRP1* gene will proliferate. Leu^+^ transformants were checked that they had lost the *URA3* gene by confirming uracil auxotrophy in order to confirm replacement of the *URA3* cassette by the *LEU2* gene after cassette exchange. In order to assay toxicity the reporter cell containing the integrase expression plasmid or empty pCM184 were grown on SD-Trp agar with or without doxycycline (1 μg/ml) in serial tenfold dilutions starting from a starting number of 5x10^4^ . pRS313 was used as the source of the *His3* gene.

### Molecular biology

Site specific recombinants arising as a result of cassette exchange were checked by PCR, Southern blotting and sequencing as described in [6].

## Additional file

Additional file 1:
**Xu and Brown Additional file.** (PDF 4447 kb)

## Abbreviations

NLS: Nuclear localization signal
