# Supplementary material for: Comparison and optimization of ten phage encoded serine integrases for genome engineering in Saccharomyces cerevisiae
Source: BMC Biotechnol. 2016 Feb 9;16:13. doi: 10.1186/s12896-016-0241-5 (PMC4748531; doi:10.1186/s12896-016-0241-5)
Supplement: Additional file 1: — Xu and Brown Additional file. (PDF 4447 kb) [file 12896_2016_241_MOESM1_ESM.pdf]

Xu and Brown; Additional file.

Plasmids used in this work.

A complete list of the plasmids used in this work is provided in Table 1 of the Additional file. The donor construct pBS attB (array) *LEU2* attB (array) (figure 1 of main text) was constructed by cutting pBS attB CCAGneo attB (BNB) [1] with SpeI and BamHI to excise the CCAGneo fragment. The plasmid vector pBS attB attB was purified and ligated to an SpeI BamHI digested PCR fragment containing the *LEU2* gene amplified from YDpLeu [2] using primers 529/530 (Table 2 of Additional file). The assay construct pBSLeuL attP array *URA3* attP array LeuR was constructed as follows; pBS attP CCAGHyTk attP [1] was digested with AscI , the left hand LEU2 targeting fragment amplified from S. cerevisiae strain T7107 with primers 524 and 534 was similarly digested with AscI and cloned into the AscI digested pBS attP CCAGHyTk attP and oriented by PCR. The resulting plasmid was then digested with NsiI and the right hand LEU2 targeting fragment (amplified with primers 531 and 533) cloned into this site. The product of these two cloning steps was then digested with SalI and BglII to remove the CCAG HyTK gene and a fragment containing the URA3 gene amplified from YDpURA3[2] using primers 526 and 527 was ligated into the digested vector after XhoI and BglII digestion. For the construction of the integrase expression plasmids the plasmid pCM184 was digested with BamHI and NotI and purified, The integrase genes were amplified with the primers listed in table 2 of the Additional file using the codon optimized NLS containing genes described in [1] as templates , the products were digested with DpnI to remove undigested template and then ligated to the pCM184 vector using the SLiCE technique [3]. As a specific example primers 576 and 577 were used to amplify the codon optimized ϕC31 integrase with an NLS and primers 576 and 578 were used to amplify the codon optimized ϕC31 integrase without an NLS. This led to the construction of a total of 30 expression plasmids; two for each of the 15 integrases, one with the NLS and one lacking an NLS. All plasmids were checked by DNA sequencing.

Additional file figure 1; analysis of site specific integration at arrays of recombinase attachment (att) sites by PCR

A; shows PCR analysis across the recombination breakpoints of ten Leu^+ prototrophs generated by ϕC31 integrase mediated site specific recombination in the assay shown in figure 1 of the main text..

B; shows PCR analysis across the recombination breakpoints of ten Leu^+ prototrophs generated by BxB1 integrase mediated site specific recombination in the assay shown in figure 1 of the main text..

Additional file figure 2; validation of conservative and reciprocal site specific recombination by sequencing across the recombinant products

The sequence traces are labelled with the name of the recombinant sequence and below each is the corresponding predicted sequence.

Additional file figure 3; analysis of the integrity of site specific integration at arrays of recombinase attachment (att) sites by agarose gel electrophoresis and filter hybridization ("Southern blotting")

A; shows the structure and sequence organization of the *LEU2* locus containing the integrated *URA3* assay construct before and after BxB1 integrase mediated site specific recombination with the donor construct containing *LEU2.*

B; shows analysis of the structure of the locus by restriction enzyme digestion using BamHI and BclI, blotting and filter hybridization with a single *attP* array. The sizes and relative intensities of the cognate fragments are consistent with the map shown in A.

Additional file figure 4; analysis of the integrity of site specific integration at a single pair of inverted sites by long range PCR

A; shows the size and sequence organization of the PCR product generated from *URA3* assay construct integrated at the *LEU2* locus before and after site specific recombination with the *HIS3* containing donor construct.

B; shows agarose gel and restriction enzyme analysis of the PCR products generated by amplification across the LEU2 locus before and after site-specific recombination with either the BxB1 or ϕC31 integrases. Two clones isolated after recombination were analysed for each integrase.

Additional file figure 5; analysis of site specific integration at a single pair of inverted sites by PCR

A; The *attB* and *attP* sites participating in the reaction are arranged in inverted orientations (main text figure 4) and consequently the incoming His3 sequences can orient in either one of two orientations, inverted one with respect to another and to the flanking *Leu2* sequences. Given this we needed to analyse each candidate recombinant with four sets of primer pairs arranged as indicated in this diagram. The lines with arrowheads indicate primers and the numbers refer to the primers used in the PCRs analysed in B.

B; shows the results of the analyses for the ϕC31 and BxB1 recombinants and demonstrates that indeed the products of the site specific integration reactions are arranged in either one of two orientations with respect to the flanking *Leu2* gene

Additional file Table S1; sequences of attachment sites

Integrase	*attB*	*attP*
ϕC31	tgcgggtgccagggcgtgcccttgggctccccgggcgcgtactcc	gtgccccaactggggtaacctttgagttctctcagttgggg
R4	gcgcccaagttgcccatgaccatgccgaagcagtggtagaagggcaccggcagacac	aggcatgttccccaaagcgataccacttgaagcagtggtactgcttgtgggtacactctgcgggtgatga
ϕBT1	gtccttgaccaggtttttgacgaaagtgatccagatgatccagctccacaccccgaacgc	ggtgctgggttgttgtctctggacagtgatccatgggaaactactcagcaccaccaatgttcc
BxB	tcggccggcttgtcgacgacggcggtctccgtcgtcaggatcatccgggc	gtcgtggtttgtctggtcaaccaccgcggtctcagtggtgtacggtacaaaccccgac
TG1	gatcagctccgcgggcaagaccttctccttcacggggtggaaggtc	tcaaccccgttccagcccaacagtgttagtctttgctcttacccagttgggcgggatagcctgcccg
ϕC1	aacgattttcaaaggatcactgaatcaaaagtattgctcatccacgcgaaattttc	aatattttaggtatatgattttgtttattagtgtaaataacactatgtacctaaaat
ϕC370	tgtaaaggagactgataatggcatgtacaactatactcgtcggtaaaaaggca	taaaaaaatacagcgtttttcatgtacaactatactagttgtagtgcctaaa
ϕK38	gagcgccggatcagggagtggacggcctgggagcgctacacgctgtggctgcggtc	ccctaatacgcaagtcgataactctcctgggagcgttgacaacttgcgcaccctga
RV	tctcgtggtggtggaaggtgttggtgcggggttggccgtggtcgaggtggggtggtggtagccattcg	gcacaggtgtagtgtatctcacaggtccacggttggccgtggactgctgaagaacattccacgccagga
SPBC	agtgcagcatgtcattaatatcagtacagataaagctgtatctcctgtgaacacaatgggtgcca	aaagtagtaagtatcttaaaaacagataaagctgtatattaagatacttactac
TP901	tgataattgccaacacaattaacatctcaatcaaggtaaatgctttttcgtttt	aattgcgagtttttatttcgtttatttcaattaaggtaactaaaaaactccttt
Wβ	aaggtagcgtcaacgataggtgtaactgtcgtgtttgtaacggtacttccaacagctggcgtttcagt	tagttttaaagttggttattagttactgtgatatttatcacggtacccaataaccaatgaatatttga
A118	tgtaacttttcggatcaagctatgaaggacgcaaagagggaactaaacacttaatt	ttgtttagttcctcgttttctctcgttggaagaagaagaaacgagaaactaaaatta
BL3	caacctgttgacatgtttccacagacaactcacgtggaggtagtcacggcttttacgttagtt	gagaatactgttgaacaatgaaaaactaggcatgtagaagttgtttgtgcactaactttaa
MR11	acaggtcaacacatcgcagttatcgaacaatcttcgaaaatgtatggaggcacttgtatcaatataggatgtataccttcgaagacacttgtacatgatggattagaaggcaaatccttt	caaaataaaaaacattgattttattaacttcttttgtgcggaactacgaacagttcattaatacgaagtgtacaaacttccatacaaaaataaccacgacaattaagacgtggtttcta

Additional file Table S2 Additional file; plasmids used in this work

**Plasmid**
Assay construct (pBSLeuL-attP array-URA3-attP array-LeuR)
Donor construct (pBSattB array-Leu2-attB array)
Integrase expression constructs:
1). pCM184COφC31nls+
2). pCM184COφC31nls-
3). pCM184COBxB1nls+
4). pCM184COBxB1nls-
5). pCM184COφBT1nls+
6). pCM184COφBT1nls-
7). pCM184COWβnls+
8). pCM184COWβnls-
9). pCM184COTP901nls+
10). pCM184COTP901nls-
11). pCM184COTG1nls+
12). pCM184COTG1nls-
13). pCM184COSPBCnls+
14). pCM184COSPBCnls-
15). pCM184COR4nls+
16). pCM184COR4nls-
17). pCM184CORVnls+
18). pCM184CORVnls-
19). pCM184COMR11nls+
20). pCM184COMR11nls-
21). pCM184COφ370nls+
22). pCM184COφ370nls-
23). pCM184COA118nls+
24). pCM184COA118nls-
25). pCM184COφC1nls+
26). pCM184COφC1nls-
27). pCM184COφK38nls+
28). pCM184COφK38nls-
29). pCM184COBL3nls+
30). pCM184COBL3nls-

Additional file Table S3 Additional file; primers used in plasmid construction

number	name	sequence
524	AscPPLeutargetingLR	gatc ggcgcgcc ggatcc tcacaatacttgaagttgacaa
534	Asc PPLeutargetingLF	gatc ggcgcgcc TAG AAT GGT ATA TCC TTG AA
531	NsiPPLeutargetingRF-2	gatc atgcat TCC TTG CTT AAA AAG ATT CTC
533	Nsi PPLeutargetingRR-2	gatc atgcat ggatcc GAT TGA TTG AGC CTA CCC TAT G
526	XhoPPURAF	gatc ctcgag ggtgattgattgagcaagctg
527	BglURAR	gatc agatct CGG TGA TTG ATT GAG CAA GCT
529	SpeBBLeuF	GATC actagtTTCCCGGGGATCCGGTGATT
530	BsmBBLeuR	gatc gaatgca CTAGCTTGGCTGCAGGTCGA
537	BT1F	acaaatacacacactaaattaccggatcaattcgggg atgtcacctttattgcaccc
538	BT1R+nls	ATT ACA TGA TGC GGC CCT CCT GCA GGG CCC TAG CG TCA GAC TTT CCG TTT CTT CTT GGG C
539	BT1R-nls	ATT ACA TGA TGC GGC CCT CCT GCA GGG CCC TAG CG tca CAG GGC AGC CAG TTC TCT CTC
540	WBetaF	acaaatacacacactaaattaccggatcaattcgggg atgaaatacgcagtctacgtc
541	WbetaR+nls	ATT ACA TGA TGC GGC CCT CCT GCA GGG CCC TAG CG TCA GAC TTT CCG CTT TTT CTT TGG
542	WbetaR-nls	ATT ACA TGA TGC GGC CCT CCT GCA GGG CCC TAG Cgtca CAG GCT GAA GGT GTA TTC GAT
543	TP901F	acaaatacacacactaaattaccggatcaattcgggg atgactaaaaaggtcgctatc
544	TP901R+nls	ATT ACA TGA TGC GGC CCT CCT GCA GGG CCC TAG CG TCA GAC CTT CCG CTT CTT CTT TG
545	TP901R-nls	ATT ACA TGA TGC GGC CCT CCT GCA GGG CCC TAG CG tca TGC CAG CTG AAA CTT GAA AAT AAT G
546	TG1F	acaaatacacacactaaattaccggatcaattcgggg atgtccgtgaaagtcgagggc
547	TG1R+nls	ATT ACA TGA TGC GGC CCT CCT GCA GGG CCC TAG CG TCA CAC CTT CCG TTT CTT CTT AG
548	TG1R-nls	ATT ACA TGA TGC GGC CCT CCT GCA GGG CCC TAG CG tca GGC AGC GGC AGT AAA GCC ATT C
549	SPBCF	acaaatacacacactaaattaccggatcaattcgggg atggagctgaagaacatcgtc
550	SPBCR+nls	ATT ACA TGA TGC GGC CCT CCT GCA GGG CCC TAG CG TCA GAC TTT GCG TTT CTT TTT TG
551	SPBCR-nls	ATT ACA TGA TGC GGC CCT CCT GCA GGG CCC TAG CG TCA GTG GAA GCT GTT AGT TGC TGT C
552	RVF	acaaatacacacactaaattaccggatcaattcgggg atgagatacaccacaccagtg
553	RVR+nls	ATT ACA TGA TGC GGC CCT CCT GCA GGG CCC TAG CG TCA GAC CTT CCG CTT CTT CT
554	RVR-nls	ATT ACA TGA TGC GGC CCT CCT GCA GGG CCC TAG CG TCA CCG CCA GTT GAC CTG CAC

555	R4F	acaaatacacacactaaattaccggatcaattcgggg atgaacagaggaggaccaacag
556	R4+nls	ATT ACA TGA TGC GGC CCT CCT GCA GGG CCC TAG cg TCA CAC TTT TCT TTT CTT TTT AG
557	R4-nls	ATT ACA TGA TGC GGC CCT CCT GCA GGG CCC TAG cg tca CTC TGC CAC ATC TCT CCA CTC
558	MR11F	acaaatacacacactaaattaccggatcaattcgggg atgaaggtcgccatctacacc
559	MR11R+nls	ATT ACA TGA TGC GGC CCT CCT GCA GGG CCC TAG cg TCA GAC CTT GCG TTT CTT CTT G
560	MR11R-nls	ATT ACA TGA TGC GGC CCT CCT GCA GGG CCC TAG cgtca GTA AAA GTC GAT GTT CTT AAT C
561	phi370F	acaaatacacacactaaattaccggatcaattcgggg atgagaaaagtcgccatctac
562	phi370R+nls	ATT ACA TGA TGC GGC CCT CCT GCA GGG CCC TAG cg TCA GAC CTT CCG CTT CTT TTT G
563	phi370R-nls	ATT ACA TGA TGC GGC CCT CCT GCA GGG CCC TAG cg TCA TGC CAG CTG AAA CTT GAA AAT G
564	BL3F	acaaatacacacactaaattaccggatcaattcgggg atgaagctgagggccgctatc
565	BL3R+nls	ATT ACA TGA TGC GGC CCT CCT GCA GGG CCC TAG cg TCA CAC CTT CCG CTT TTT CTT TG
566	BL3R-nls	ATT ACA TGA TGC GGC CCT CCT GCA GGG CCC TAG cgtca GAT GTT CCA TTC AAT CTT CAT G
567	A118F	acaaatacacacactaaattaccggatcaattcgggg atgaaagccgctatctacatc
568	A118R+nls	ATT ACA TGA TGC GGC CCT CCT GCA GGG CCC TAG cg TCA CAC CTT TCT CTT TTT CTT GG
569	A118R-nls	ATT ACA TGA TGC GGC CCT CCT GCA GGG CCC TAG cg TCA CAG CCA CTC AAT GGT CAC C
570	FC1F	acaaatacacacactaaattaccggatcaattcgggg atgaagcgtgcagcattgtata
571	FC1R+nls	ATT ACA TGA TGC GGC CCT CCT GCA GGG CCC TAG cg CTA CAC CTT GCG CTT CTT CTT G
572	FC1R-nls	ATT ACA TGA TGC GGC CCT CCT GCA GGG CCC TAG cg TCA AAA TTT ATA TTT AAT AAT TAC CTC
573	K38F	acaaatacacacactaaattaccggatcaattcgggg atgtggtcccacccccagttc
574	K38R+nls	ATT ACA TGA TGC GGC CCT CCT GCA GGG CCC TAG cg CTA CAC CTT GCG CTT CTT C
575	K38R-nls	ATT ACA TGA TGC GGC CCT CCT GCA GGG CCC TAG cg TCA CGT CCT CGC CGC CCA TTT G
576	FC31F	acaaatacacacactaaattaccggatcaattcgggg atggatacctacgccggag
577	FC31R+nls	ATT ACA TGA TGC GGC CCT CCT GCA GGG CCC TAG cg TCA CAC TTT CCG CTT TTT CTT AG
578	FC31R-nls	ATT ACA TGA TGC GGC CCT CCT GCA GGG CCC TAG cg TCA GGC GGC CAC GTC CTC GGT G

622	LeuTF BT1 attP URAF	tttacatttcagcaatatatatatatatatttcaaggatataccattcta ggtgctgggttgttgtctctggacagtgatccatgggaaactactcagcaccacc aatgttcc tgattgattgagcaagctggg
623	LeuTRBT1 attP URAR	ATT ATG AAT TTC ATT TAT AAA GTT TAT GTA CAA ATA TCA TAA AAA AAG AGA ATC TTT TTA GGTGCTGGGTTGTTGTCTCTGGACAGTGATCCATGGGAAACTACTCAGCACCACC AATGTTCC GT GAT TGA TTG AGC AAG CTG
644	BT1attB His3 F	gatc GTCCTTGACCAGGTTTTTGACGAAAGTGATCCAGATGATCCAGCTCCACACCCCG AACGC attccgttttaagagcttggtgag
645	BT1 attB His3 R	gatc GTCCTTGACCAGGTTTTTGACGAAAGTGATCCAGATGATCCAGCTCCACACCCCG AACGC CAT ATG ATC CGT CGA GTT CAA GAG
630	LeuTF BXB attP URAF	tttacatttcagcaatatatatatatatatttcaaggatataccattcta GTCGTGGTTTGTCTGGTCAACCACCGCGGTCTCAGTGGTGTACGGTACAAACCCC GAC tgattgattgagcaagctggg
631	LeuTR BXB attP URAR	ATT ATG AAT TTC ATT TAT AAA GTT TAT GTA CAA ATA TCA TAA AAA AAG AGA ATC TTT TTA GTCGTGGTTTGTCTGGTCAACCACCGCGGTCTCAGTGGTGTACGGTACAAACCCC GAC GT GAT TGA TTG AGC AAG CTG
642	BxBattB His3 F	gatcTCGGCCGGCTTGTCGACGACGGCGGTCTCCGTCGTCAGGATCATCCGGGCa ttccgttttaagagcttggtgag
643	BxBattB His3 R	gatcTCGGCCGGCTTGTCGACGACGGCGGTCTCCGTCGTCAGGATCATCCGGGC CAT ATG ATC CGT CGA GTT CAA GAG
592	LeuTFatP URAF	tttacatttcagcaatatatatatatatatttcaaggatataccattcta GTAGTGCCCCAACTGGGGTAACCTTTGAGTTCTCTCAGTTGGGGGCGTAGtgatt gattgagcaagctggg
593	LeuTRattP URAR	ATT ATG AAT TTC ATT TAT AAA GTT TAT GTA CAA ATA TCA TAA AAA AAG AGA ATC TTT TTA GTAGTGCCCCAACTGGGGTAACCTTTGAGTTCTCTCAGTTGGGGGCGTAGGT GAT TGA TTG AGC AAG CTG
640	Leu2R-3	CTT CTT GAT AAA TGT ATG TAG ATT G
641	Leu2F-3	ctatttctcaacaagtaattggttg
650	His1289F	tatacgtgtcattctgaacgagg
651	His 241R	TTC AAA CGA TAC CTG GCA GTG AC
646	FC31 attB His3 F	gatc CGGTGCGGGTGCCAGGGCGTGCCCTTGGGCTCCCCGGGCGCGTACTCCAC attccgttttaagagcttggtgag
647	FC31 attB His3 R	gatc CGGTGCGGGTGCCAGGGCGTGCCCTTGGGCTCCCCGGGCGCGTACTCCAC CAT ATG ATC CGT CGA GTT CAA GAG
648	HR His3F	tttcttacctttacatttcagcaatatatatatatatatttcaaggatatacca ttcta attccgttttaagagcttggtgag
649	HR His3R	ATT ATG AAT TTC ATT TAT AAA GTT TAT GTA CAA ATA TCA TAA AAA AAG AGA ATC TTT TTA CAT ATG ATC CGT CGA GTT CAA GAG

Additional file Table S4 Additional file: confirmation of the accuracy of site-specific recombination mediated by serine integrases at single recombination sites

Integrase	PCR for attR-1 (PCR +ve number /number checked)	PCR for attR-2 (PCR +ve number / number checked)
**ϕC31**	10/10;10/10;10/10	10/10;10/10;10/10
**ϕBT1**	10/10;10/10;10/10	10/10;10/10;10/10
**BxB**	10/10;10/10;10/10	10/10;10/10;10/10

The table shows the number of site site-specific recombination reactions that yielded a specific PCR product expressed as a proportion of those analysed.

References

1. Xu Z, Thomas L, Davies B, Chalmers R, Smith M, Brown W: **Accuracy and efficiency define Bxb1 integrase as the best of fifteen candidate serine recombinases for the integration of DNA into the human genome**. *BMC Biotechnol* 2013, **13**:87.
2. Berben G, Dumont J, Gilliquet V, Bolle PA, Hilger F: **The YDp plasmids: a uniform set of vectors bearing versatile gene disruption cassettes for Saccharomyces cerevisiae**. *Yeast* 1991, **7**(5):475-477.
3. Zhang Y, Werling U, Edelmann W: **SLiCE: a novel bacterial cell extract-based DNA cloning method**. *Nucleic Acids Res* 2012, **40**(8):e55.

Xu and Brown, additional file figure S1

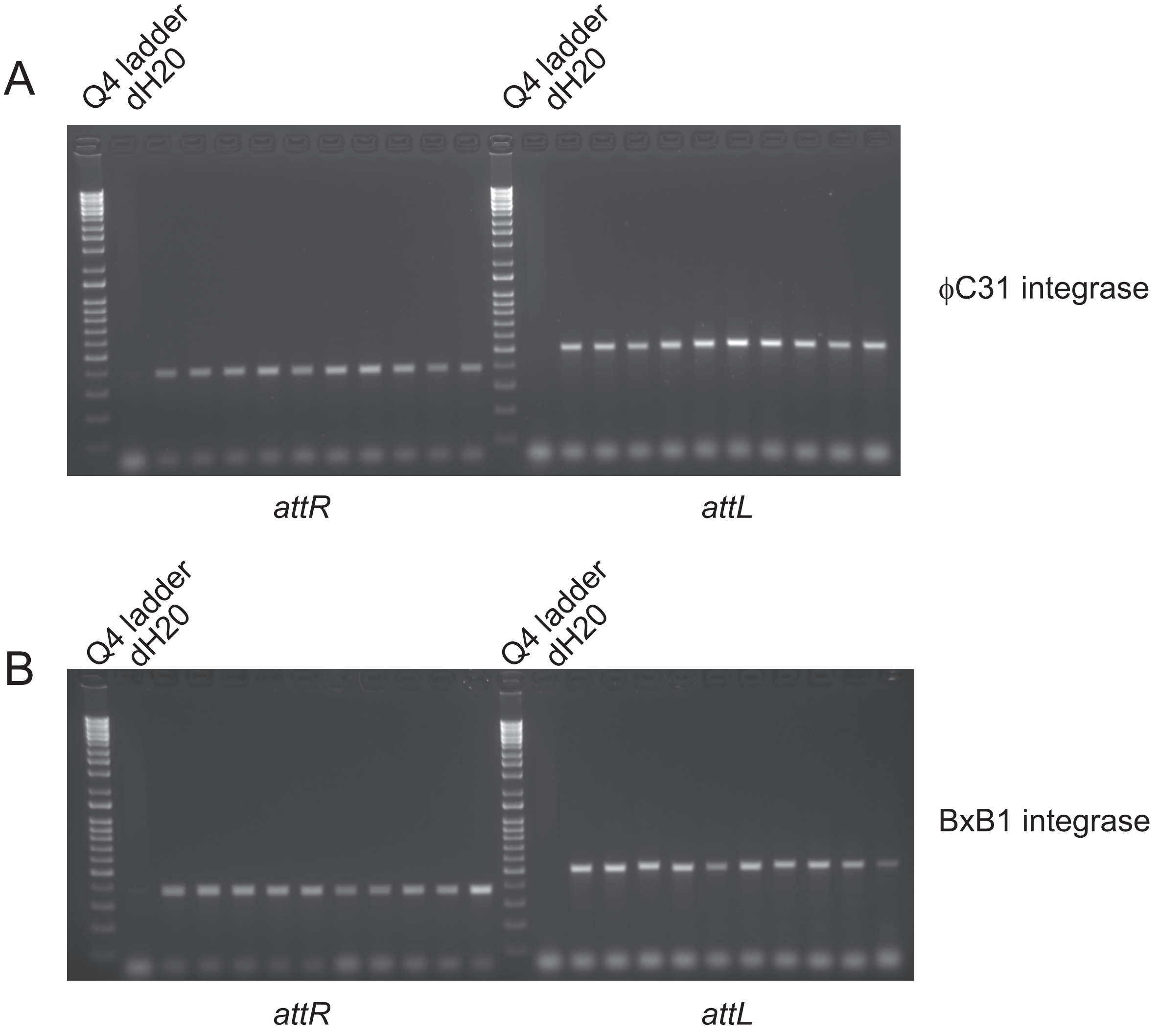

Xu and Brown, additional file figure S2

BxB1 *attL*

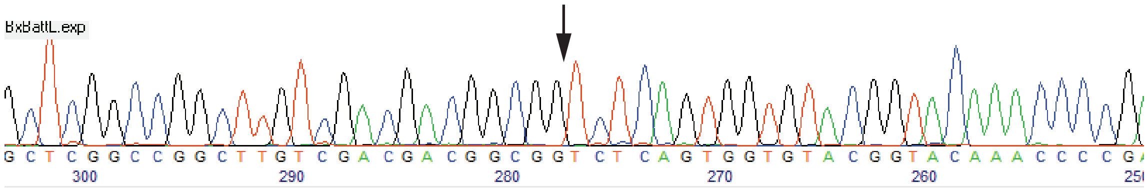

TCGGCCGGCTTGTCGACGACGGCG**GT**CTCAGTGGTGTACGGTACAAACCCCGAC

BxB1 *attR*

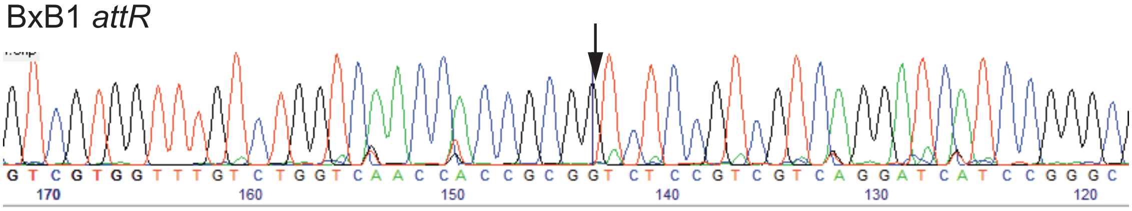

GTCGTGGTTTGTCTGGTCAACCACCGCG**GT**CTCCGTCGTCAGGATCATCCGGGC

ϕBT1 *attL*

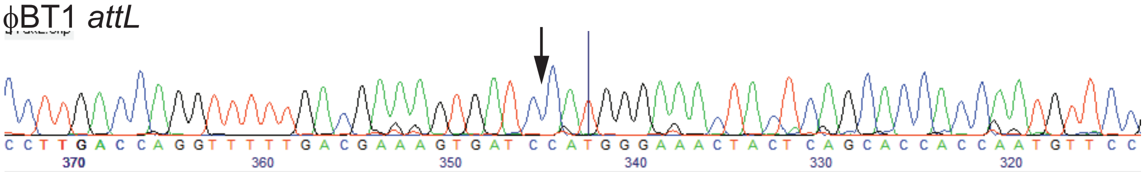

GTCCTTGACCAGGTTTTTGACGAAAGTGAT**CC**ATGGGAAACTACTCAGCACCACCAATGTTCC

ϕBT1 *attR*

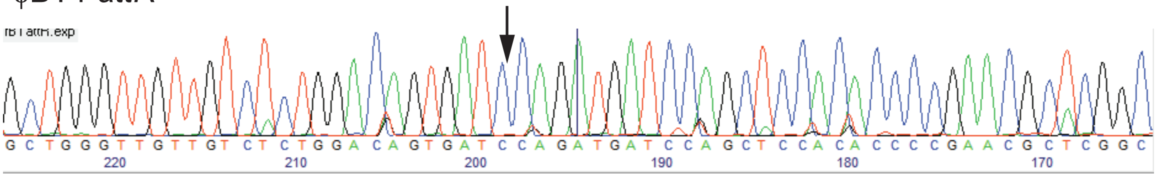

GCTGGGTTGTTGTCTCTGGACAGTGAT**CC**AGATGATCCAGCTCCACACCCCGAACGCTCGGC

R4 *attL*

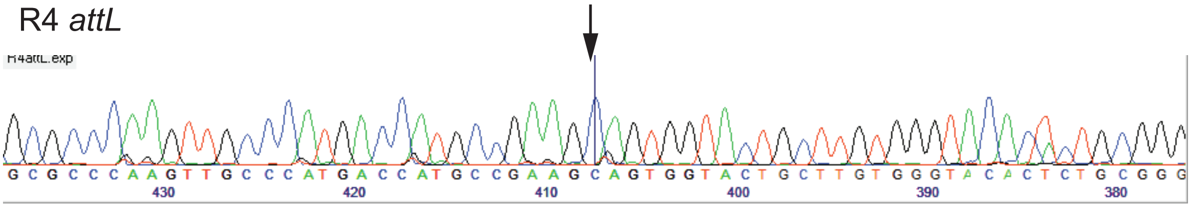

GCGCCCAAGTTGCCCATGACCATGCC**GAAGCAGTGGTA**CTGCTTGTGGGTACACTCTGCGGGTGATGA

R4 *attR*

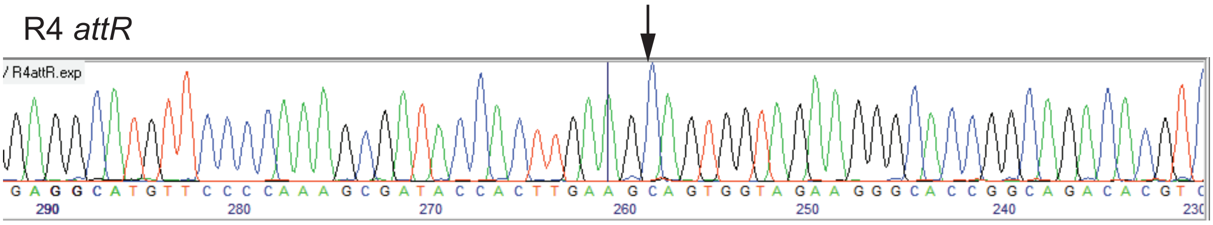

AGGCATGTTCCCCAAAGCGATACCACTT**GAAGCAGTGGTA**GAAGGGCACCGGCAGACAC

Xu and Brown, additional file figure S3

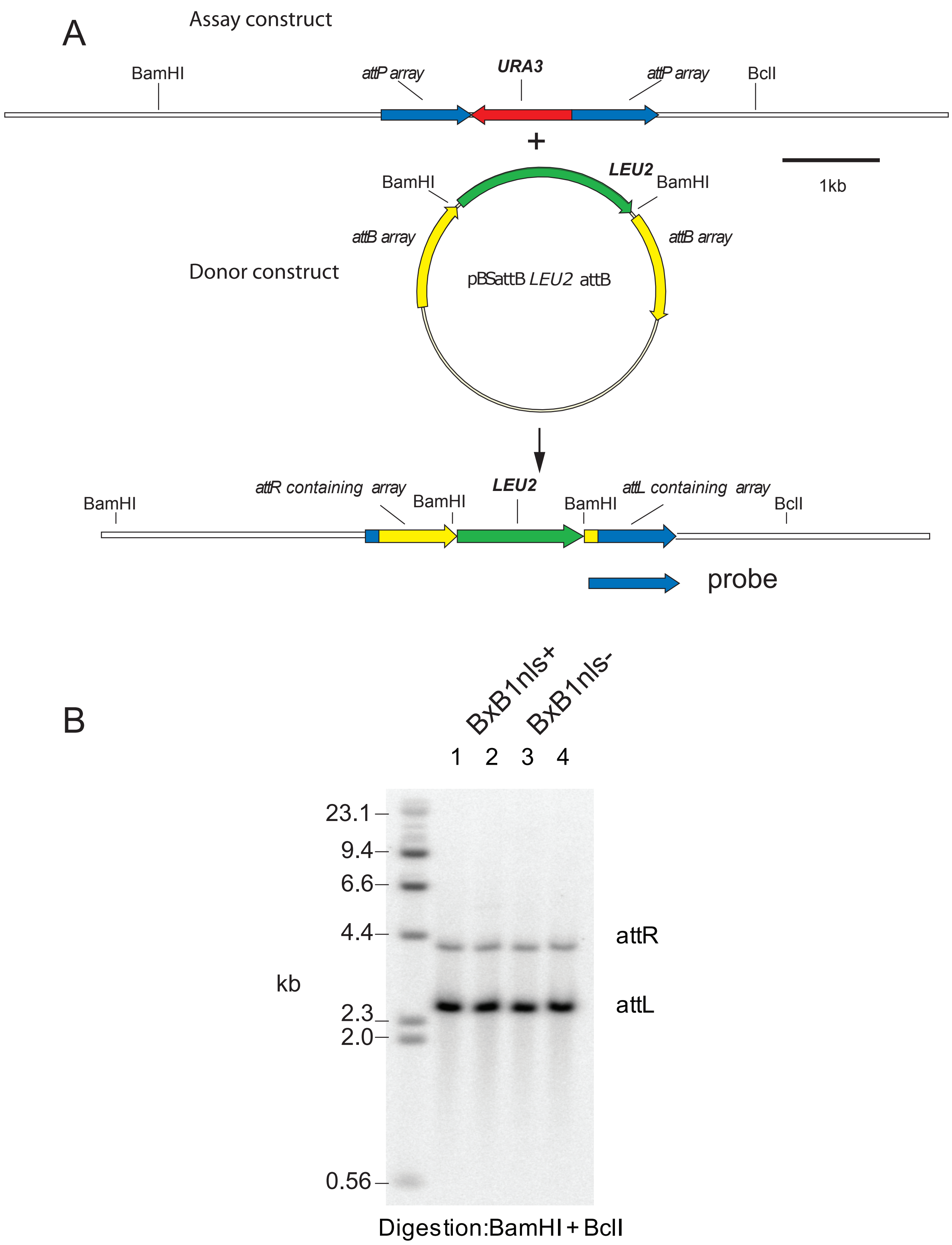

Xu and Brown, additional file figure S4

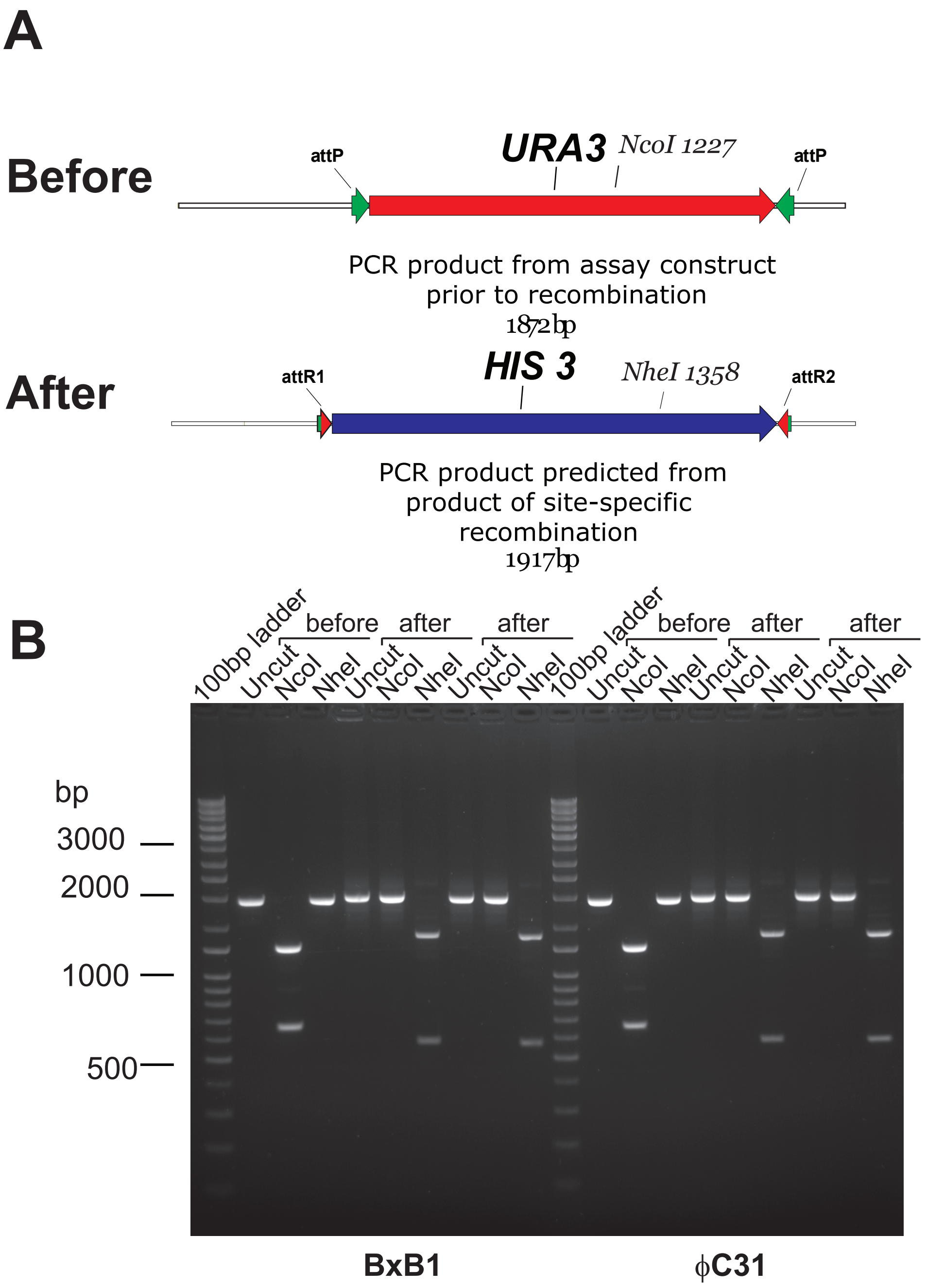

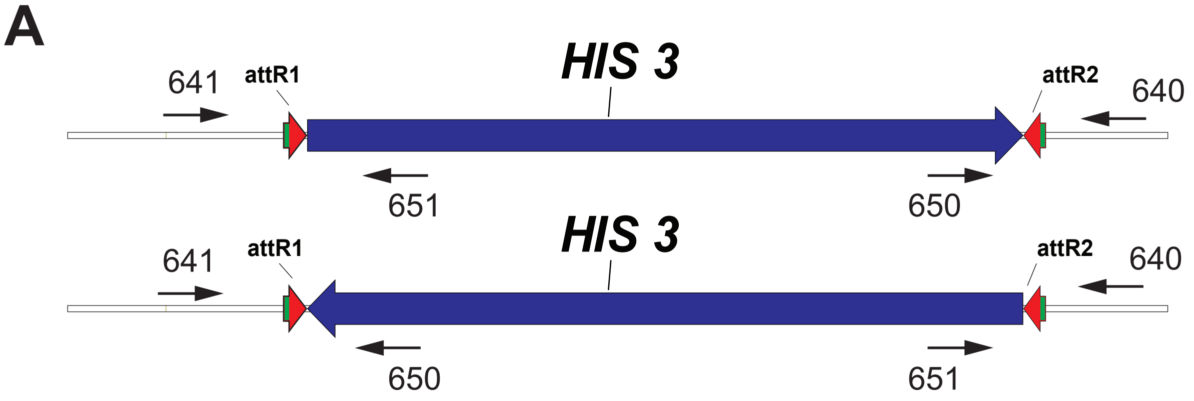
A
HIS 3
641
attR1
attR2
640
651
650
HIS 3
641
attR1
attR2
640
650
651

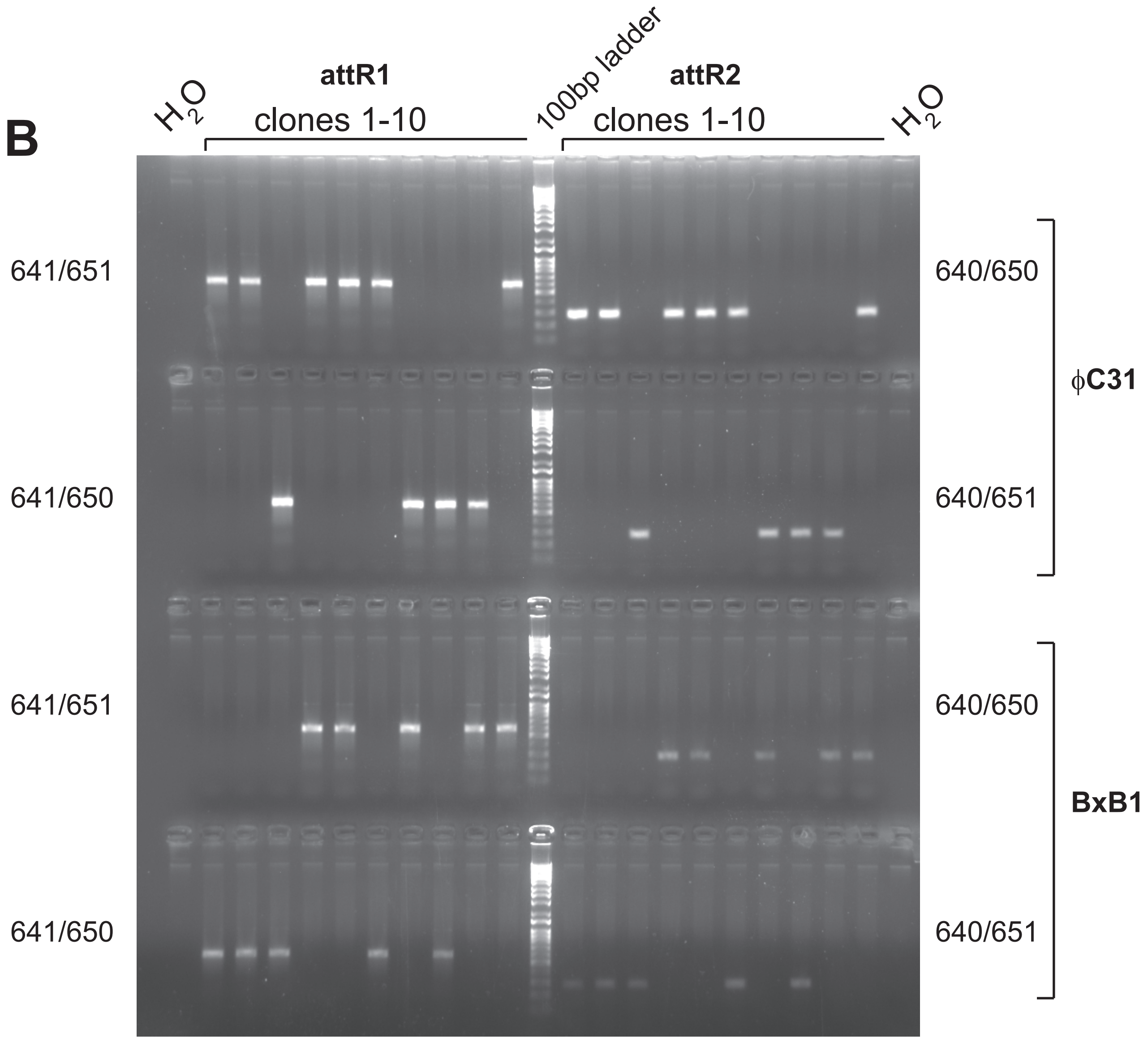
B
H2O
attR1
clones 1-10
100bp ladder
attR2
clones 1-10
H2O
641/651
641/650
641/651
641/650
640/650
640/651
640/650
640/651
φC31
BxB1
